# Ethyl 2-(5-fluoro-3-methyl­sulfinyl-1-benzofuran-2-yl)acetate

**DOI:** 10.1107/S1600536809025938

**Published:** 2009-07-11

**Authors:** Hong Dae Choi, Pil Ja Seo, Byeng Wha Son, Uk Lee

**Affiliations:** a Department of Chemistry, Dongeui University, San 24 Kaya-dong Busanjin-gu, Busan 614-714, Republic of Korea; b Department of Chemistry, Pukyong National University, 599-1 Daeyeon 3-dong, Nam-gu, Busan 608-737, Republic of Korea

## Abstract

In the title compound, C_13_H_13_FO_4_S, the O atom and the methyl group of the methyl­sulfinyl substituent lie on opposite sides of the plane through the benzofuran fragment. The crystal structure exhibits four inter­molecular non-classical C—H⋯O hydrogen bonds. In addition, the crystal structure contains aromatic π–π inter­actions between the furan and benzene rings of adjacent mol­ecules [centroid–centroid distance = 3.743 (2) Å], and two inter­molecular C—H⋯π inter­actions.

## Related literature

For the crystal structures of similar ethyl 2-(5-halo-3-methyl­sulfinyl-1-benzofuran-2-yl)acetate derivatives. see: Choi *et al.* (2007*a* ▶,*b* ▶,*c* ▶). For the pharmacological activity of benzofuran compounds, see: Howlett *et al.* (1999 ▶); Twyman & Allsop (1999 ▶). 
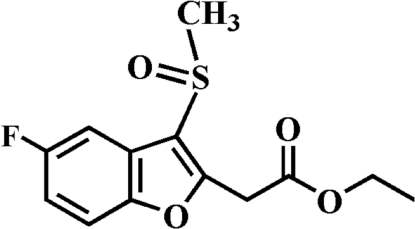


## Experimental

### 

#### Crystal data

C_13_H_13_FO_4_S
*M* *_r_* = 284.29
Triclinic, 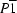

*a* = 7.8821 (5) Å
*b* = 9.0922 (5) Å
*c* = 10.4354 (6) Å
α = 73.682 (1)°
β = 79.155 (1)°
γ = 66.622 (1)°
*V* = 656.31 (7) Å^3^
*Z* = 2
Mo *K*α radiation
μ = 0.27 mm^−1^
*T* = 273 K
0.40 × 0.40 × 0.10 mm

#### Data collection

Bruker SMART CCD diffractometer
Absorption correction: none
5673 measured reflections
2805 independent reflections
2512 reflections with *I* > 2σ(*I*)
*R*_int_ = 0.063

#### Refinement

*R*[*F* ^2^ > 2σ(*F* ^2^)] = 0.036
*wR*(*F* ^2^) = 0.101
*S* = 1.04
2805 reflections
173 parameters
H-atom parameters constrained
Δρ_max_ = 0.40 e Å^−3^
Δρ_min_ = −0.35 e Å^−3^

### 

Data collection: *SMART* (Bruker, 2001 ▶); cell refinement: *SAINT* (Bruker, 2001 ▶); data reduction: *SAINT*; program(s) used to solve structure: *SHELXS97* (Sheldrick, 2008 ▶); program(s) used to refine structure: *SHELXL97* (Sheldrick, 2008 ▶); molecular graphics: *ORTEP-3* (Farrugia, 1997 ▶) and *DIAMOND* (Brandenburg, 1998 ▶); software used to prepare material for publication: *SHELXL97*.

## Supplementary Material

Crystal structure: contains datablocks global, I. DOI: 10.1107/S1600536809025938/ez2175sup1.cif

Structure factors: contains datablocks I. DOI: 10.1107/S1600536809025938/ez2175Isup2.hkl

Additional supplementary materials:  crystallographic information; 3D view; checkCIF report

## Figures and Tables

**Table 1:** Hydrogen-bond geometry (Å, °)

*D*—H⋯*A*	*D*—H	H⋯*A*	*D*⋯*A*	*D*—H⋯*A*
C3—H3⋯O4^i^	0.93	2.42	3.3374 (19)	168
C5—H5⋯O3^ii^	0.93	2.67	3.482 (2)	147
C9—H9*A*⋯O4^iii^	0.97	2.21	3.177 (2)	172
C9—H9*B*⋯O1^iv^	0.97	2.59	3.542 (2)	169
C11—H11*A*⋯*Cg*2^v^	0.97	2.92	3.773 (2)	148
C12—H12*C*⋯*Cg*1^v^	0.97	2.81	3.502 (2)	129

Symmetry codes: (i) 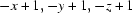; (ii) 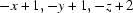; (iii) 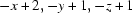; (iv) 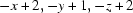; (v) 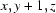.*Cg*1 and *Cg*2 are the centroids of the C1/C2/C7/O1/C8 furan ring and the C2–C7 benzene ring, respectively.

**Fig. 1.**
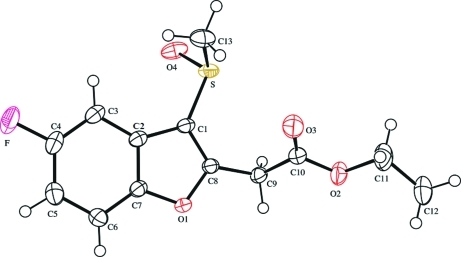
The molecular structure of the title compound with the atom numbering scheme. Displacement ellipsoids are drawn at the 50% probability level. H atoms are presented as a small cycles of arbitrary radius.

**Fig. 2.**
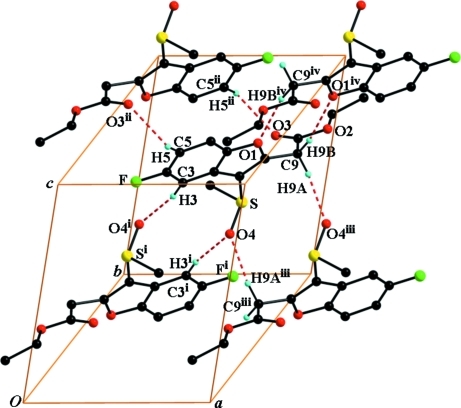
The C–H···O interactions (dotted lines) in the title compound. [Symmetry code: (i) - x + 1, - y + 1, - z + 1; (ii) - x + 1, - y + 1, - z + 2; (iii) - x + 2, - y + 1, - z + 1; (iv) - x + 2, - y + 1, - z + 2.]

**Fig. 3.**
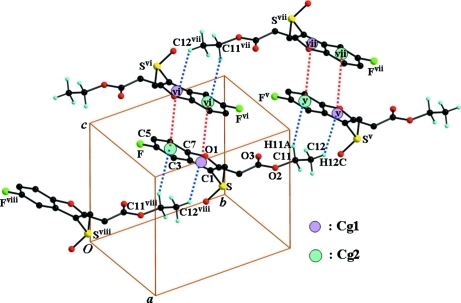
The π–π and C–H···π interactions (dotted lines) in the title compound. Cg denotes the ring centroids. [Symmetry code: (v) x, 1 + y, z; (vi) 1 - x, 1 - y, 2 - z; (vii) 1 - x, 2 - y, 2 - z; (viii) x, - 1 + y, z.]

## supplementary crystallographic information

### Comment

Molecules containing the benzofuran ring system have attracted considerable interest in view of their biological and pharmacological properties (Howlett *et al.*, 1999; Twyman & Allsop, 1999). This work is related to our communications on the synthesis and structures of ethyl 2-(5-halo-3-methylsulfinyl-1-benzofuran-2-yl)acetate analogues, viz. ethyl 2-(5-chloro-3-methylsulfinyl-1-benzofuran-2-yl)acetate (Choi *et al.*, 2007*a*), ethyl 2-(5-bromo-3-methylsulfinyl-1-benzofuran-2-yl) acetate (Choi *et al.*, 2007*b*), and ethyl 2-(5-iodo-3-methylsulfinyl-1-benzofuran-2-yl)acetate (Choi *et al.*,  2007*c*). Here we report the crystal structure of the title compound (Fig. 1).

The benzofuran unit is essentially planar, with a mean deviation of 0.010 (1) Å from the least-squares plane defined by the nine constituent atoms. The crystal packing (Fig. 2) exhibits weak intermolecular C–H···O non-classical hydrogen bonds; the first between an H atom of the benzofuran ring and the S═O unit, with a C3–H3···O4^i^, the second between an H atom of benzofuran ring and the C═O unit, with a C5-H5···O3^ii^, the third between an H atom of the methylene group bonded to carboxylate C atom and the S═O unit, with a C9–H9A···O4^iii^, the fourth between an H atom of the methylene group bonded to carboxylate C atom and the furan O atom, with a C9–H9B···O1^iv^, respectively (Table 1 and Fig. 2). Additionally, the crystal packing (Fig. 3) contains aromatic π–π interactions between the furan and the benzene rings of the neighbouring molecules, with a Cg1···Cg2^vi^ distance of 3.743 (2) Å (Cg1 and Cg2 are the centroids of the C1/C2/C7/O1/C8 furan ring and the C2-C7 benzene ring, respectively). The molecular packing is further stabilized by two intermolecular C–H···π interactions; the first between the methylene H atom of ethoxy group and the benzene ring of a neighbouring molecule (C11–H11A···Cg2^v^), the second between the methyl H atom of ethoxy group and the furan ring of a neighbouring molecule (C12–H12C···Cg1^v^), respectively (Table 1 and Fig. 3).

### Experimental

77% 3-chloroperoxybenzoic acid (247 mg, 1.1 mmol) was added in small portions to a stirred solution of ethyl 2-(5-fluoro-3-methylsulfanyl-1-benzofuran-2-yl)acetate (268 mg, 1.0 mmol) in dichloromethane (30 ml) at 273 K. After being stirred for 4 h at room temperature, the mixture was washed with saturated sodium bicarbonate solution and the organic layer was separated, dried over magnesium sulfate, filtered and concentrated in vacuum. The residue was purified by column chromatography (hexane-ethyl acetate,1:2 v/v) to afford the title compound as a colorless solid [yield 79%, m.p. 401-402 K; *R*_f_ = 0.43 (hexane-ethyl acetate, 1;2 v/v )]. Single crystals suitable for X-ray diffraction were prepared by evaporation of a solution of the title compound in acetone at room temperature.

### Refinement

All H atoms were geometrically positioned and refined using a riding model, with C-H = 0.93 Å for the aryl, 0.97 Å for the methylene, and 0.96 Å for the methyl H atoms. *U*_iso_(H) = 1.2*U*_eq_(C) for the aryl and methylene H atoms, and 1.5*U*_eq_(C) for methyl H atoms.

### Crystal data

**Table:**

C_13_H_13_FO_4_S	*Z* = 2
*M**_r_* = 284.29	*F*(000) = 296
Triclinic, *P*1	*D*_x_ = 1.439 Mg m^−^^3^
Hall symbol: -P 1	Mo *K*α radiation, λ = 0.71073 Å
*a* = 7.8821 (5) Å	Cell parameters from 4105 reflections
*b* = 9.0922 (5) Å	θ = 2.5–27.5°
*c* = 10.4354 (6) Å	µ = 0.27 mm^−^^1^
α = 73.682 (1)°	*T* = 273 K
β = 79.155 (1)°	Block, colourless
γ = 66.622 (1)°	0.40 × 0.40 × 0.10 mm
*V* = 656.31 (7) Å^3^	

### Data collection

**Table:**

Bruker SMART CCD diffractometer	2512 reflections with *I* > 2σ(*I*)
Radiation source: fine-focus sealed tube	*R*_int_ = 0.063
graphite	θ_max_ = 27.0°, θ_min_ = 2.0°
Detector resolution: 10.0 pixels mm^-1^	*h* = −10→10
φ and ω scans	*k* = −11→11
5673 measured reflections	*l* = −13→13
2805 independent reflections	

### Refinement

**Table:**

Refinement on *F*^2^	Primary atom site location: structure-invariant direct methods
Least-squares matrix: full	Secondary atom site location: difference Fourier map
*R*[*F*^2^ > 2σ(*F*^2^)] = 0.036	Hydrogen site location: difference Fourier map
*wR*(*F*^2^) = 0.101	H-atom parameters constrained
*S* = 1.04	*w* = 1/[σ^2^(*F*_o_^2^) + (0.0465*P*)^2^ + 0.2336*P*] where *P* = (*F*_o_^2^ + 2*F*_c_^2^)/3
2805 reflections	(Δ/σ)_max_ < 0.001
173 parameters	Δρ_max_ = 0.40 e Å^−^^3^
0 restraints	Δρ_min_ = −0.34 e Å^−^^3^

### Special details

**Table:**

Geometry. All esds (except the esd in the dihedral angle between two l.s. planes) are estimated using the full covariance matrix. The cell esds are taken into account individually in the estimation of esds in distances, angles and torsion angles; correlations between esds in cell parameters are only used when they are defined by crystal symmetry. An approximate (isotropic) treatment of cell esds is used for estimating esds involving l.s. planes.
Refinement. Refinement of F^2^ against ALL reflections. The weighted R-factor wR and goodness of fit S are based on F^2^, conventional R-factors R are based on F, with F set to zero for negative F^2^. The threshold expression of F^2^ > 2sigma(F^2^) is used only for calculating R-factors(gt) etc. and is not relevant to the choice of reflections for refinement. R-factors based on F^2^ are statistically about twice as large as those based on F, and R- factors based on ALL data will be even larger.

### Fractional atomic coordinates and isotropic or equivalent isotropic displacement parameters (Å^2^)

**Table:**

	*x*	*y*	*z*	*U*_iso_*/*U*_eq_	
S	0.72668 (5)	0.63168 (5)	0.54574 (3)	0.02919 (13)	
F	0.32177 (16)	0.21796 (16)	0.88301 (12)	0.0529 (3)	
O1	0.84077 (14)	0.45046 (13)	0.92200 (9)	0.0247 (2)	
O2	1.01504 (16)	0.88467 (14)	0.73052 (13)	0.0368 (3)	
O3	0.73837 (17)	0.89429 (16)	0.69571 (14)	0.0428 (3)	
O4	0.74634 (18)	0.50040 (17)	0.47717 (11)	0.0422 (3)	
C7	0.7124 (2)	0.38103 (18)	0.92490 (14)	0.0246 (3)	
C1	0.7324 (2)	0.53985 (18)	0.71812 (13)	0.0235 (3)	
C2	0.6385 (2)	0.43348 (18)	0.80110 (14)	0.0236 (3)	
C3	0.5036 (2)	0.3785 (2)	0.78410 (16)	0.0296 (3)	
H3	0.4504	0.4109	0.7035	0.036*	
C4	0.4545 (2)	0.2735 (2)	0.89391 (18)	0.0342 (4)	
C5	0.5303 (2)	0.2183 (2)	1.01700 (17)	0.0350 (4)	
H5	0.4925	0.1450	1.0866	0.042*	
C6	0.6629 (2)	0.2741 (2)	1.03413 (15)	0.0301 (3)	
H6	0.7157	0.2412	1.1149	0.036*	
C8	0.8506 (2)	0.54532 (18)	0.79478 (13)	0.0228 (3)	
C9	0.9836 (2)	0.63165 (18)	0.76654 (14)	0.0254 (3)	
H9A	1.0746	0.5942	0.6946	0.030*	
H9B	1.0489	0.6012	0.8456	0.030*	
C10	0.8940 (2)	0.81678 (19)	0.72758 (14)	0.0268 (3)	
C11	0.9500 (3)	1.0649 (2)	0.6923 (2)	0.0499 (5)	
H11A	0.8265	1.1120	0.7346	0.060*	
H11B	0.9456	1.1029	0.5959	0.060*	
C12	1.0827 (3)	1.1155 (2)	0.7373 (2)	0.0458 (4)	
H12A	1.2045	1.0677	0.6952	0.055*	
H12B	1.0850	1.0781	0.8329	0.055*	
H12C	1.0441	1.2333	0.7130	0.055*	
C13	0.4872 (2)	0.7641 (2)	0.54508 (17)	0.0385 (4)	
H13A	0.4622	0.8305	0.4563	0.058*	
H13B	0.4603	0.8341	0.6059	0.058*	
H13C	0.4108	0.6988	0.5727	0.058*	

### Atomic displacement parameters (Å^2^)

**Table:**

	*U*^11^	*U*^22^	*U*^33^	*U*^12^	*U*^13^	*U*^23^
S	0.0300 (2)	0.0380 (3)	0.01935 (19)	−0.01155 (17)	−0.00708 (14)	−0.00428 (15)
F	0.0482 (6)	0.0562 (8)	0.0708 (8)	−0.0359 (6)	−0.0137 (6)	−0.0093 (6)
O1	0.0298 (5)	0.0263 (5)	0.0213 (5)	−0.0122 (4)	−0.0090 (4)	−0.0032 (4)
O2	0.0328 (6)	0.0229 (6)	0.0573 (7)	−0.0104 (5)	−0.0141 (5)	−0.0059 (5)
O3	0.0347 (6)	0.0308 (7)	0.0608 (8)	−0.0108 (5)	−0.0202 (6)	0.0016 (6)
O4	0.0430 (7)	0.0561 (9)	0.0291 (6)	−0.0093 (6)	−0.0085 (5)	−0.0218 (5)
C7	0.0259 (7)	0.0238 (7)	0.0261 (7)	−0.0083 (6)	−0.0063 (5)	−0.0073 (5)
C1	0.0262 (7)	0.0247 (7)	0.0208 (6)	−0.0073 (6)	−0.0067 (5)	−0.0065 (5)
C2	0.0241 (7)	0.0229 (7)	0.0245 (7)	−0.0057 (6)	−0.0058 (5)	−0.0082 (5)
C3	0.0274 (7)	0.0308 (8)	0.0346 (8)	−0.0092 (6)	−0.0089 (6)	−0.0118 (6)
C4	0.0297 (8)	0.0324 (9)	0.0480 (9)	−0.0156 (7)	−0.0057 (7)	−0.0126 (7)
C5	0.0360 (9)	0.0291 (8)	0.0397 (9)	−0.0154 (7)	−0.0015 (7)	−0.0035 (7)
C6	0.0349 (8)	0.0271 (8)	0.0275 (7)	−0.0114 (7)	−0.0063 (6)	−0.0024 (6)
C8	0.0259 (7)	0.0219 (7)	0.0209 (6)	−0.0072 (6)	−0.0056 (5)	−0.0050 (5)
C9	0.0248 (7)	0.0252 (8)	0.0275 (7)	−0.0086 (6)	−0.0077 (5)	−0.0052 (5)
C10	0.0285 (7)	0.0274 (8)	0.0258 (7)	−0.0112 (6)	−0.0058 (6)	−0.0043 (6)
C11	0.0485 (11)	0.0228 (9)	0.0790 (14)	−0.0104 (8)	−0.0232 (10)	−0.0047 (9)
C12	0.0414 (10)	0.0269 (9)	0.0701 (13)	−0.0142 (8)	−0.0037 (9)	−0.0109 (8)
C13	0.0342 (8)	0.0366 (10)	0.0365 (9)	−0.0027 (7)	−0.0147 (7)	−0.0031 (7)

### Geometric parameters (Å, °)

**Table:**

S—O4	1.5007 (13)	C5—C6	1.387 (2)
S—C1	1.7583 (14)	C5—H5	0.9300
S—C13	1.7914 (18)	C6—H6	0.9300
F—C4	1.3633 (17)	C8—C9	1.4859 (19)
O1—C8	1.3723 (17)	C9—C10	1.509 (2)
O1—C7	1.3813 (16)	C9—H9A	0.9700
O2—C10	1.3343 (17)	C9—H9B	0.9700
O2—C11	1.466 (2)	C11—C12	1.488 (3)
O3—C10	1.2035 (19)	C11—H11A	0.9700
C7—C6	1.381 (2)	C11—H11B	0.9700
C7—C2	1.3972 (19)	C12—H12A	0.9600
C1—C8	1.3603 (19)	C12—H12B	0.9600
C1—C2	1.446 (2)	C12—H12C	0.9600
C2—C3	1.3984 (19)	C13—H13A	0.9600
C3—C4	1.373 (2)	C13—H13B	0.9600
C3—H3	0.9300	C13—H13C	0.9600
C4—C5	1.396 (2)		
			
O4—S—C1	106.41 (7)	O1—C8—C9	116.26 (11)
O4—S—C13	106.04 (8)	C8—C9—C10	113.99 (12)
C1—S—C13	98.86 (8)	C8—C9—H9A	108.8
C8—O1—C7	106.33 (10)	C10—C9—H9A	108.8
C10—O2—C11	116.58 (13)	C8—C9—H9B	108.8
C6—C7—O1	125.26 (12)	C10—C9—H9B	108.8
C6—C7—C2	124.13 (13)	H9A—C9—H9B	107.6
O1—C7—C2	110.62 (12)	O3—C10—O2	123.99 (15)
C8—C1—C2	107.22 (12)	O3—C10—C9	126.12 (13)
C8—C1—S	122.68 (11)	O2—C10—C9	109.88 (12)
C2—C1—S	129.73 (10)	O2—C11—C12	107.55 (15)
C7—C2—C3	119.44 (13)	O2—C11—H11A	110.2
C7—C2—C1	104.72 (12)	C12—C11—H11A	110.2
C3—C2—C1	135.84 (13)	O2—C11—H11B	110.2
C4—C3—C2	115.78 (14)	C12—C11—H11B	110.2
C4—C3—H3	122.1	H11A—C11—H11B	108.5
C2—C3—H3	122.1	C11—C12—H12A	109.5
F—C4—C3	118.07 (14)	C11—C12—H12B	109.5
F—C4—C5	116.90 (15)	H12A—C12—H12B	109.5
C3—C4—C5	125.04 (14)	C11—C12—H12C	109.5
C6—C5—C4	119.12 (15)	H12A—C12—H12C	109.5
C6—C5—H5	120.4	H12B—C12—H12C	109.5
C4—C5—H5	120.4	S—C13—H13A	109.5
C7—C6—C5	116.48 (14)	S—C13—H13B	109.5
C7—C6—H6	121.8	H13A—C13—H13B	109.5
C5—C6—H6	121.8	S—C13—H13C	109.5
C1—C8—O1	111.11 (12)	H13A—C13—H13C	109.5
C1—C8—C9	132.64 (13)	H13B—C13—H13C	109.5
			
C8—O1—C7—C6	178.81 (15)	F—C4—C5—C6	−178.60 (15)
C8—O1—C7—C2	−1.02 (16)	C3—C4—C5—C6	1.5 (3)
O4—S—C1—C8	127.12 (13)	O1—C7—C6—C5	179.74 (15)
C13—S—C1—C8	−123.16 (14)	C2—C7—C6—C5	−0.4 (2)
O4—S—C1—C2	−45.00 (15)	C4—C5—C6—C7	−0.8 (2)
C13—S—C1—C2	64.72 (15)	C2—C1—C8—O1	−0.08 (17)
C6—C7—C2—C3	1.0 (2)	S—C1—C8—O1	−173.75 (10)
O1—C7—C2—C3	−179.12 (13)	C2—C1—C8—C9	179.69 (15)
C6—C7—C2—C1	−178.88 (14)	S—C1—C8—C9	6.0 (2)
O1—C7—C2—C1	0.96 (16)	C7—O1—C8—C1	0.67 (16)
C8—C1—C2—C7	−0.53 (16)	C7—O1—C8—C9	−179.15 (12)
S—C1—C2—C7	172.54 (12)	C1—C8—C9—C10	61.4 (2)
C8—C1—C2—C3	179.57 (17)	O1—C8—C9—C10	−118.84 (14)
S—C1—C2—C3	−7.4 (3)	C11—O2—C10—O3	0.1 (2)
C7—C2—C3—C4	−0.4 (2)	C11—O2—C10—C9	178.79 (15)
C1—C2—C3—C4	179.54 (16)	C8—C9—C10—O3	−13.8 (2)
C2—C3—C4—F	179.21 (14)	C8—C9—C10—O2	167.56 (12)
C2—C3—C4—C5	−0.9 (3)	C10—O2—C11—C12	164.80 (15)

### Hydrogen-bond geometry (Å, °)

**Table:**

*D*—H···*A*	*D*—H	H···*A*	*D*···*A*	*D*—H···*A*
C3—H3···O4^i^	0.93	2.42	3.3374 (19)	168
C5—H5···O3^ii^	0.93	2.67	3.482 (2)	147
C9—H9A···O4^iii^	0.97	2.21	3.177 (2)	172
C9—H9B···O1^iv^	0.97	2.59	3.542 (2)	169
C11—H11A···Cg2^v^	0.97	2.92	3.773 (2)	148
C12—H12C···Cg1^v^	0.97	2.81	3.502 (2)	129

Symmetry codes: (i) −*x*+1, −*y*+1, −*z*+1; (ii) −*x*+1, −*y*+1, −*z*+2; (iii) −*x*+2, −*y*+1, −*z*+1; (iv) −*x*+2, −*y*+1, −*z*+2; (v) *x*, *y*+1, *z*.
